# The correlation between probiotic use and outcomes of cancer patients treated with immune checkpoint inhibitors

**DOI:** 10.3389/fphar.2022.937874

**Published:** 2022-08-30

**Authors:** Lilong Zhang, Qi Jin, Dongqi Chai, Tianrui Kuang, Chunlei Li, Yongjun Guan, Li Liu, Weixing Wang, Wenhong Deng

**Affiliations:** ^1^ Department of General Surgery, Renmin Hospital of Wuhan University, Wuhan, China; ^2^ Department of Neurology, The First Hospital of Jilin University, Changchun, China

**Keywords:** immune checkpoint inhibitors, probiotics, meta-analysis, non-small cell lung cancer, prognosis

## Abstract

**Objective:** Immune checkpoint inhibitors (ICIs) have recently demonstrated promising results in improving the prognosis of cancer patients. The goal of this meta-analysis was to determine the impact of probiotic use on the survival of cancer patients treated with ICIs.

**Methods:** Before 3 March 2022, the eligible literature was searched using PubMed, EMBASE, Cochrane Library, Google Scholar, and Clinical trials.gov databases. Overall survival (OS), progression-free survival (PFS), objective response rate (ORR), and disease control rate (DCR) were the primary endpoints.

**Results:** A total of 6 studies met the inclusion criteria, and 1,123 patients were included. Meta-analysis showed a trend for probiotic use to prolong PFS (HR: 0.585, 95% CI: 0.328–1.045, *p* = 0.070) and increase DCR (HR: 1.868, 95% CI: 0.890–3.922, *p* = 0.099), although it was of borderline statistical significance. We also found that probiotics significantly improved OS (HR: 0.526, 95% CI: 0.341–0.812, *p* = 0.004) and ORR (OR: 2.831, 95% CI: 1.578–5.076, *p* < 0.001) in ICI-treated cancer patients. Besides, subgroup analysis showed that non-small cell lung cancer (NSCLC) patients treated with ICIs in combination with probiotics would achieve significantly longer PFS (HR: 0.532, 95% CI: 0.354–0.798, *p* = 0.002) and OS (HR: 0.528, 95% CI: 0.306–0.912, *p* = 0.022), as well as higher ORR (OR: 2.552, 95% CI: 1.279–5.091, *p* = 0.008) and DCR (OR: 2.439, 95% CI: 1.534–3.878, *p* < 0.001). Sensitivity analysis showed that the above results are stable and reliable. The publication bias test confirmed that there was no publication bias in these results.

**Conclusion:** Current evidence reveals that probiotics can improve the efficacy of ICI treatment in NSCLC patients.

**Systematic Review Registeration:** https://www.crd.york.ac.uk/prospero/, identifier CRD42022316104.

## Introduction

Immune checkpoint inhibitors (ICIs), including anti-programmed cell death protein-(L)-1 (anti-PD-(L)1) and anti-cytotoxic T-lymphocyte-associated protein 4 (anti-CTLA-4) monoclonal antibodies, reactivate the antitumor activity of CD8^+^ T cells by blocking T cell signals and have changed the landscape of advanced cancer treatment (Ribas and Wolchok, 2018). ICIs have been approved for multiple tumors and have been shown to improve patient survival when compared to traditional treatments (Ribas and Wolchok, 2018; Tang et al., 2018). However, the clinical efficacy of ICIs varies widely amongst patients, with only a tiny percentage of patients benefiting from treatment. Furthermore, primary resistance to ICIs is still frequent, and a significant number of patients continue to worsen or relapse as a result of ICI resistance (Sharma et al., 2017; Seto et al., 2019). Regrettably, no perfect biomarkers for predicting the efficacy of ICIs exist at this time. Thus, the search for prospective biomarkers that influence its efficacy is critical for a more targeted selection of treatment populations in clinical practice.

The impact of gut microbiota on tumorigenesis and response to treatment with ICIs is receiving increasing attention. Two landmark studies in mice provided the first evidence that the gut microbiome had a direct impact on ICI effectiveness (Sivan et al., 2015; Vetizou et al., 2015). Recently, prospective studies have revealed that microbiome diversity and composition were strongly associated with the efficacy of ICIs in patients with metastatic melanoma (Gopalakrishnan et al., 2018; Matson et al., 2018; Lee et al., 2022), renal cell carcinoma (RCC) (Derosa et al., 2020; Salgia et al., 2020), and non-small cell lung cancer (NSCLC) (Huemer et al., 2019; Hakozaki et al., 2020), among others. Probiotics can change the gut microbiome, which is described as a single or combination of bacterial species that, when given in sufficient proportions, confer a health benefit to the host (Panebianco et al., 2018; Tinsley et al., 2020). In many animal studies, probiotics have been shown to help the body benefit in the treatment of ICIs (Gao et al., 2021; Lee et al., 2021; Si et al., 2022). However, the association between probiotic use and the efficacy of ICIs remains unclear in cancer patients due to a lack of comprehensive evaluation. Therefore, we conducted the first systematic review and meta-analysis to elucidate whether probiotic use affects the efficacy of ICI therapy. This will provide evidence for future clinical use of probiotics in cancer patients treated with ICIs, thereby maximizing the clinical benefit to patients.

## Methods

### Literature search strategies

This meta-analysis followed the Preferred Reporting Items for Systematic Reviews and Meta-Analyses (PRISMA) guidelines (Page et al., 2021). The protocol for this meta-analysis was available in PROSPERO (CRD42022316104). On 3 March 2022, PubMed (https://pubmed.ncbi.nlm.nih.gov/), EMBASE (https://www.embase.com/), and Cochrane Library (https://www.cochranelibrary.com/) were retrieved. “Immune Checkpoint Inhibitors” [Mesh], "Probiotics” [Mesh], and their entry terms were searched in [All Fields]. Detailed search strategies are shown in Supplementary Table S1. We also searched Google Scholar to uncover gray literature that was not indexed in the previously listed databases, such as presentations, abstracts, and unpublished research data. An ongoing research search was undertaken on the Clinical Trial Registration Platform (https://clinicaltrials.gov/). In addition, we also manually retrieved the reference lists of eligible papers.

### Study selection criteria

If articles matched all the following criteria, they were included. 1) Patients diagnosed with cancer; 2) Patients treated with ICIs (anti-PD-(L)1 and/or anti-CTLA-4); 3) Patients separated into the non-probiotic group and probiotic group; 4) Provided at least one of the outcomes of interest (Overall survival (OS), progression-free survival (PFS), objective response rate (ORR), and disease control rate (DCR)); 5) Prospective or retrospective study. Only the article with the most comprehensive data and rigorous methods was chosen when studies reported overlapping patient populations. Meanwhile, the following exclusion criteria were employed: abstract, comments, and case report.

### Data extraction and quality assessment

Data extraction mainly focused on the author, publication year, study region, study period, study type, cancer type, the number of patients, the age of patients, the number of male patients, types of ICI treatment, and the outcomes of interest (OS, PFS, ORR, DCR). Response Evaluation Criteria in Solid Tumors (RECIST) version 1.1 was used to estimate the ORR. Complete response, partial response, or stable disease (SD) lasting longer than 6 months was considered disease control. When the hazard ratio (HR) for OS or PFS was calculated using both univariate and multivariate analyses, the multivariate analysis was favored due to confounding factor correction. If the appropriate data were not instantly accessible from published articles, authors would be attached personally to the findings. The Revised Cochrane risk-of-bias tool for randomized trials (RoB 2) (Sterne et al., 2019) was applied to estimate the methodological quality of the prospective articles. The Newcastle-Ottawa Scale (NOS) score was used to estimate the quality of the retrospective studies (Wells et al., 2019). Literature with a score ≥7 was considered to be of high quality. Three authors (Zhang Lilong, Jin Qi, and Chai Dongqi) independently cross-checked all the above steps, and the senior authors (Wenhong Deng and Wang Weixing) addressed any disparities.

### Statistical methods

Stata SE15.0 was used for the statistical analysis. The HR and its 95% confidence interval (95% CI) were used to calculate the influence of probiotic use on the risk of survival in cancer patients. The association between ICI efficacy and probiotic usage was expressed as an odds ratio (OR) with a 95% CI. The statistical heterogeneity among the studies was determined using the chi-squared test. I^2^ ≥ 50% indicates high heterogeneity, 20% ≤ I^2^ <50% suggests moderate heterogeneity, and I^2^ < 20% indicates low heterogeneity. To ensure the reliability of the results, indicators with high or moderate heterogeneity were combined using a random-effects model, while outcomes with low heterogeneity were combined using a fixed-effects model. To reduce the influence of heterogeneity on the meta-analysis, a subgroup analysis was performed. Begg’s and Egger’s tests were implemented to assess publication bias. The sensitivity analysis by the leave-one-out method was conducted to estimate the stability of the results. All *p* values were two-sided with significance set at *p* < 0.05.

## Results

### Studies retrieved and characteristics

**FIGURE 1:**
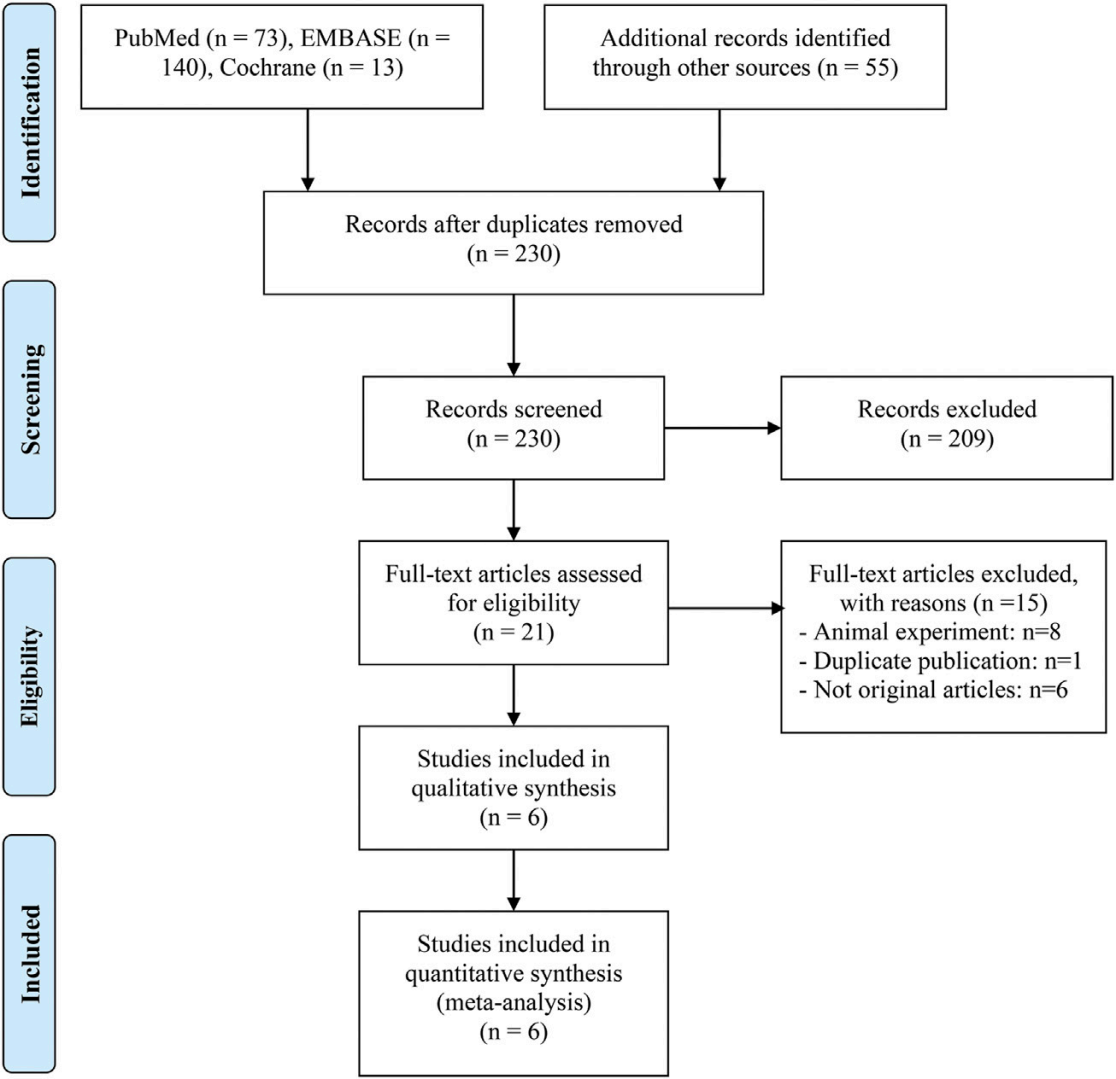
The flow diagram of identifying eligible studies.

We gathered 281 potentially eligible records and assessed their titles and abstracts to see if they were suitable for inclusion. We discovered that 6 studies (Svaton et al., 2020; Tomita et al., 2020; Miura et al., 2021; Spencer et al., 2021; Takada et al., 2021; Dizman et al., 2022) met our inclusion criteria after carefully reading the full texts of 21 records. Figure 1 depicts the flow diagram for identifying eligible studies. Table 1 shows the baseline characteristics of the included studies as well as the quality evaluation. Of the five retrospective studies, four articles were awarded 7 or 8 points and were regarded as high quality. One was rated with 6 points and was deemed as medium quality. Besides, a prospective study was considered at low risk of bias.

**TABLE 1:** Baseline characteristics of included studies.

Author, year	Study region	Study period	Study type	Cancer type	Patients with/without probiotics			Types of ICI treatment	Quality
					Number of patients	Median/mean age	Male		
Dizman et al. (2022)	United Statea	04/2019–12/2020	Prospective	mRCC	19/10	66/64	13/8	Nivolumab and ipilimumab	Low risk
Spencer et al. (2021)	United Statea	—	Retrospective	Melanoma	49/109	63.5/64	27/67	Anti-PD1 and/or anti-CTLA4	7
Tomita et al. (2020)	Japan	01/2016–05/2019	Retrospective	NSCLC	39/79	68/67	33/66	Nivolumab or pembrolizumab or atezolizumab	8
Miura et al. (2021)	Japan	01/2016–07/2018	Retrospective	NSCLC	14/286	65	226	Nivolumab or pembrolizumab	6
Takada et al. (2021)	Japan	01/2016–09/2018	Retrospective	NSCLC	32/262	67	25/208	Nivolumab or pembrolizumab	7
Svaton et al. (2020)	Czech Republic	2015–2019	Retrospective	NSCLC	6/218	65	133	Nivolumab	6

mRCC, metastatic renal cell carcinoma; NSCLC, non–small cell lung cancer; ICI, immune checkpoint inhibitor; PD1, programmed cell death 1; CTLA4, cytotoxic T-lymphocyte-associated protein 4.

### Progression-free survival

**FIGURE 2:**
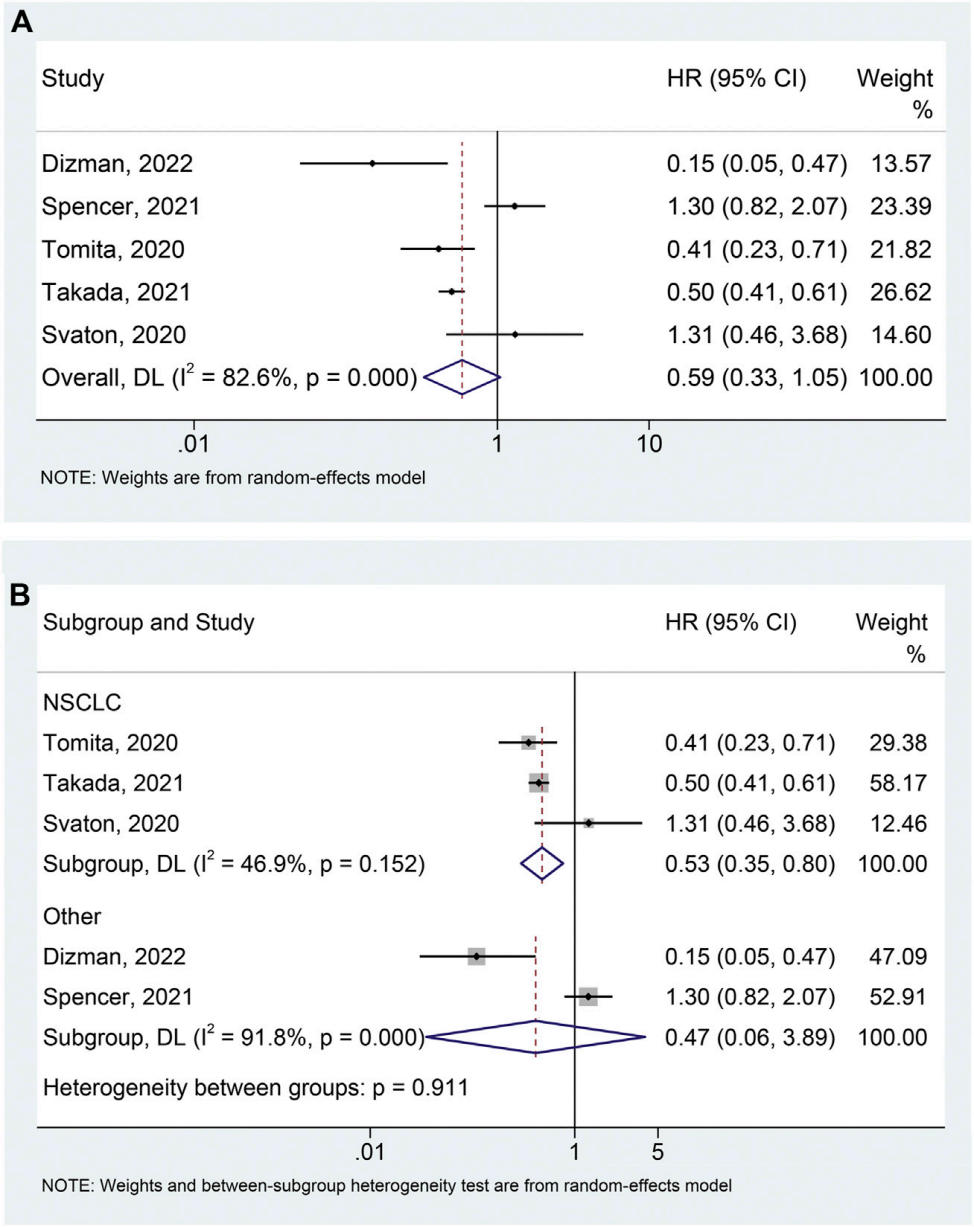
Forest plots of HR for correlation of probiotic administration with progression-free survival **(A)**. Subgroup analysis of progression-free survival based on cancer types **(B)**. NSCLC, non-small cell lung cancer; HR, hazard ratio; CL, confidence interval; Univariate analysis (Dizman et al., 2022); Multivariable analysis (Svaton et al., 2020; Tomita et al., 2020; Spencer et al., 2021; Takada et al., 2021).

The correlation between probiotic use and PFS was assessed using prognostic data from five studies (Svaton et al., 2020; Tomita et al., 2020; Spencer et al., 2021; Takada et al., 2021; Dizman et al., 2022) involving 823 participants (145 who received probiotics and 678 who did not). Due to significant heterogeneity (I^2^ = 82.6%, *p* < 0.001), we applied a random-effects model. The results showed that probiotic usage was associated with a decreased risk of poor PFS in cancer patients treated with ICIs, although it was of borderline statistical significance (HR: 0.585, 95% CI: 0.328–1.045, *p* = 0.070) (Figure 2A). The Begg’s and Egger’s tests were then performed to investigate publication bias, with the results indicating that there was no significant publication bias in our findings (Begg’s test *p* = 0.806, Egger’s test *p* = 0.840). To assess the impact of each study on the overall meta-analysis, we implemented a sensitivity analysis via the leave-one-out method. The findings revealed that no single study had a substantial impact on the pooled HR of PFS, validating the reliability of our findings (Supplementary Figure S1).

Based on the heterogeneity test, we performed a subgroup analysis to lessen the effect of heterogeneity on the pooled results. We divided the studies into two groups according to tumor type and found that the use of probiotics significantly reduced the risk of progression in patients with NSCLC (Figure 2B; I^2^ = 46.9%, *p* = 0.152; HR: 0.532, 95% CI: 0.354–0.798, *p* = 0.002), while not affecting PFS in patients with other tumors (mRCC and melanoma) (Figure 2B; I^2^ = 91.8%, *p* < 0.001; HR: 0.470, 95% CI: 0.057–3.889, *p* = 0.484). Furthermore, there was no publication bias (Begg’s test *p* = 1.000, Egger’s test *p* = 0.650) of meta-analysis results in the NSCLC patients.

### Overall survival

**FIGURE 3:**
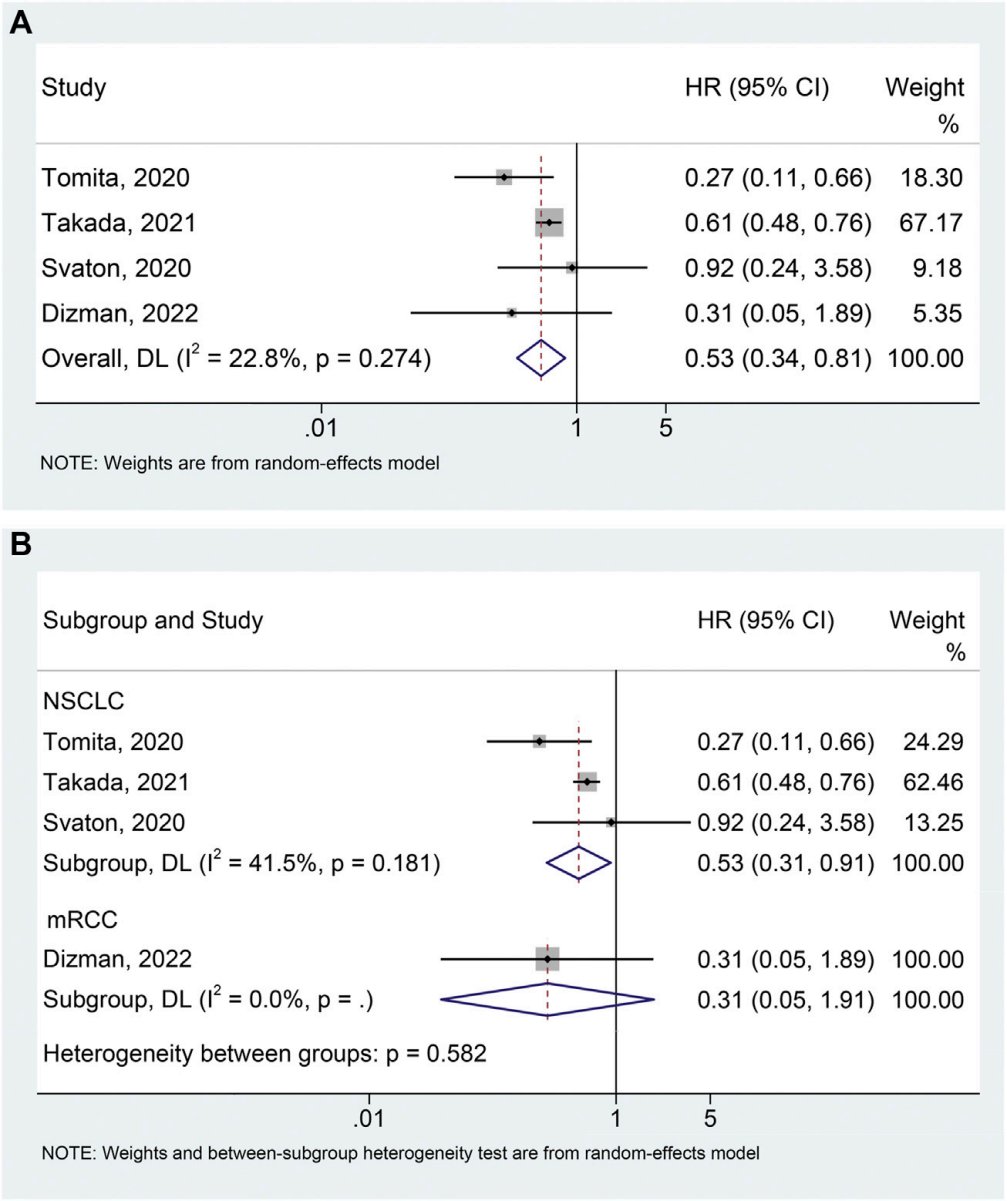
Forest plots of HR for the relationship of probiotic use with overall survival **(A)**. Subgroup analysis of overall survival based on cancer types **(B)**. NSCLC, non-small cell lung cancer; mRCC, metastatic renal cell carcinoma; HR, hazard ratio; CL, confidence interval; Univariate analysis (Dizman et al., 2022); Multivariable analysis (Svaton et al., 2020; Tomita et al., 2020; Takada et al., 2021).

The meta-analysis of OS was conducted using survival data from 4 studies (Svaton et al., 2020; Tomita et al., 2020; Takada et al., 2021; Dizman et al., 2022) with a total of 665 participants (96 with probiotics versus 569 without probiotics). As shown in Figure 3A, there was moderate heterogeneity among studies (I^2^ = 22.8%, *p* = 0.274), so a random-effects model was used. The results revealed that probiotic use was significantly related to better OS (HR: 0.526, 95% CI: 0.341–0.812, *p* = 0.004). Begg’s and Egger’s tests showed no publication bias in the meta-analysis (Begg’s test *p* = 0.734, Egger’s test *p* = 0.516). The results of the sensitivity analysis also confirmed that no single study could significantly affect the pooled HR of OS (Supplementary Figure S2). Thus, our result above was stable and reliable. Finally, we also conducted subgroup analyses to examine whether different cancer types had an impact on the outcome. Similar to the data for PFS, probiotic use was significantly associated with better OS in NSCLC patients (Figure 3B; I^2^ = 41.5%, *p* = 0.181; HR: 0.528, 95% CI: 0.306–0.912, *p* = 0.022). Besides, no significant publication bias (Begg’s test *p* = 1.000, Egger’s test *p* = 0.761) of meta-analysis results was observed in the NSCLC patients.

### Objective response rate

**FIGURE 4:**
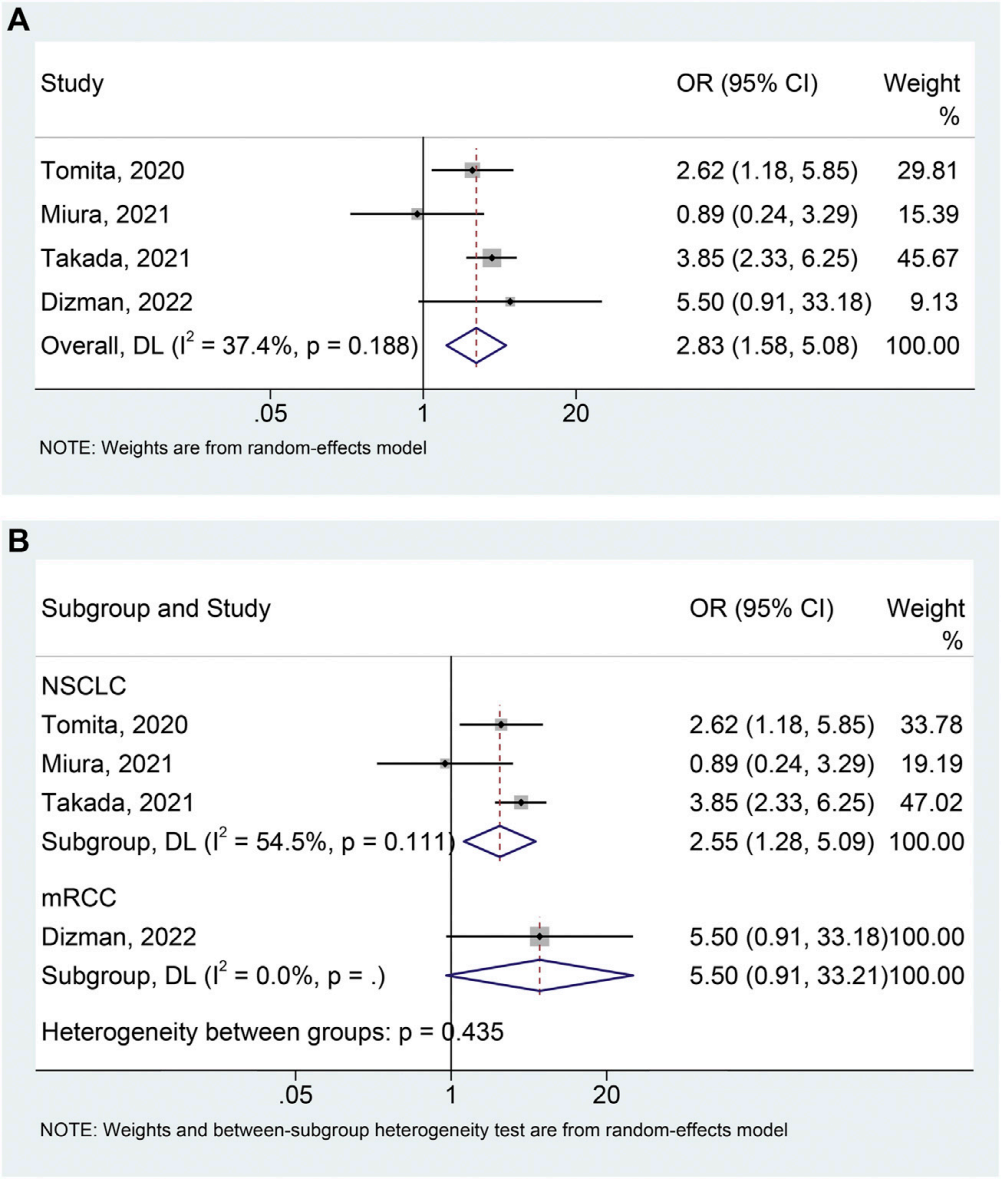
Forest plots of OR for the association of probiotic usage with objective response rate **(A)**. Subgroup analysis of the objective response rate based on cancer types **(B)**. NSCLC, non-small cell lung cancer; mRCC, metastatic renal cell carcinoma; OR, odds ratio; CL, confidence interval; Univariate analysis (Tomita et al., 2020; Dizman et al., 2022); Multivariable analysis (Miura et al., 2021; Takada et al., 2021).

Four studies (Tomita et al., 2020; Miura et al., 2021; Takada et al., 2021; Dizman et al., 2022) with 741 patients (104 with probiotics versus 637 without probiotics) were included in the meta-analysis of ORR. A random-effects model was applied due to the presence of moderate heterogeneity (I^2^ = 37.4%, *p* = 0.188). We found that the use of probiotics significantly increased the ORR (Figure 4A; OR: 2.831, 95% CI:1.578–5.076, *p* < 0.001). No remarkable publication bias was observed via the Begg’s (*p* = 0.734) and Egger’s tests (*p* = 0.535). Sensitivity analysis also revealed that the pooled results for ORR should be considered stable (Supplementary Figure S3). In addition, we conducted a subgroup analysis to explore the effect of probiotic use on ORR in NSCLC patients and the results were consistent with the above findings (Figure 4B; I^2^ = 54.5%, *p* = 0.111; OR: 2.552, 95% CI: 1.279–5.091, *p* = 0.008). There was no significant publication bias (Begg’s test *p* = 0.296, Egger’s test *p* = 0.101) of meta-analysis results in the NSCLC patients.

### Disease control rate

**FIGURE 5:**
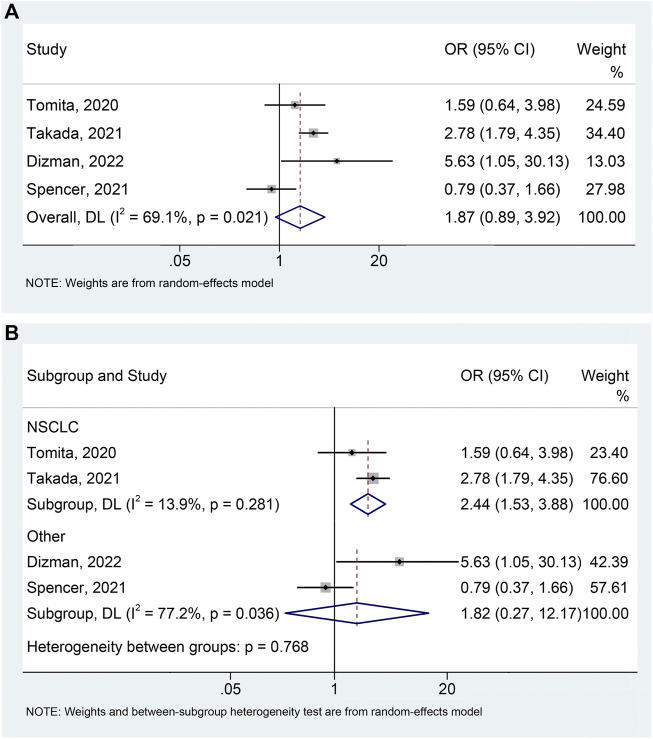
Forest plots of OR for the relationship of probiotic use with disease control rate **(A)**. Subgroup analysis of the disease control rate based on cancer types **(B)**. NSCLC, non-small cell lung cancer; OR, odds ratio; CL, confidence interval; Univariate analysis (Tomita et al., 2020; Dizman et al., 2022); Multivariable analysis (Spencer et al., 2021; Takada et al., 2021).

The meta-analysis of DCR includes 4 studies (Tomita et al., 2020; Spencer et al., 2021; Takada et al., 2021; Dizman et al., 2022) with 599 patients (139 with probiotics versus 460 without probiotics). An apparent heterogeneity was observed among the included studies (I^2^ = 69.1%, *p* = 0.021), and a random-effects model was performed. We found that cancer patients using probiotics were more likely to benefit during ICIs treatment, although it was of borderline statistical significance (Figure 5A; HR: 1.868, 95% CI: 0.890–3.922, *p* = 0.099). No remarkable publication biases were observed utilizing the Begg’s (*p* = 1.000) and Egger’s tests (*p* = 0.847). The result of sensitivity analysis demonstrated no single study was able to significantly influence the pooled results (Supplementary Figure S4). Moreover, subgroup analysis demonstrated that probiotic use significantly improved DCR in NSCLC patients treated with ICIs (I^2^ = 13.9%, *p* = 0.281; OR: 2.439, 95% CI: 1.534–3.878, *p* < 0.001) (Figure 5B), without impacting DCR in patients with other tumors (mRCC and melanoma) (Figure 5B; I^2^ = 77.2%, *p* = 0.036; OR: 1.816, 95% CI: 0.271–12.170, *p* = 0.539).

### Ongoing interventional clinical studies

Our search of clinicaltrials. gov identified seven studies currently enrolling cancer patients to explore the relationship between probiotic use and ICI efficacy (Table 2). Of these, there are two studies in NSCLC, two studies in RCC, and one each in liver cancer and bladder urothelial carcinoma. Most of these studies will be completed by 2023.

**TABLE 2:** Ongoing interventional clinical studies in cancer patients who have received/will receive ICI and probiotics.

ClinicalTrials. gov identifier	Status	Study title	Conditions	Arms and interventions	Phase	Study completion date
NCT03829111	Recruiting	CBM588, Nivolumab, and Ipilimumab in Treating patients with stage IV or advanced kidney cancer	Renal cell carcinoma	Arm1 (nivolumab + ipilimumab) vs. Arm2 (CBM588 + nivolumab + ipilimumab)	Phase 1	11 June 2023
NCT04699721	Recruiting	Clinical study of neoadjuvant chemotherapy and Immunotherapy combined with probiotics in patients with potential/resectable NSCLC	Non-small cell Lung cancer stage III	Arm1 (nivolumab + Paclitaxel + Carboplatin AUC5) vs. Arm2 (nivolumab + Paclitaxel + Carboplatin AUC5 + Bifidobacterium trifidum live powder)	Phase 1	December 2027
NCT05122546	Recruiting	CBM588 in Combination With nivolumab and cabozantinib for the treatment of advanced or metastatic kidney cancer	Renal cell carcinoma	Arm1 (nivolumab + cabozantinib S-malate) vs. Arm2 (CBM588 + nivolumab + cabozantinib S-malate)	Phase 1	30 November 2023
NCT05032014	Recruiting	Probiotics Enhance the treatment of PD-1 Inhibitors in patients with LmRCC, metastatic renal cell carcinoma; NSCLC, non–small cell lung cancer; ICI, immune checkpoint inhibitor; PD1, programmed cell death 1; CTLA4, cytotoxic T-lymphocyte-associated protein liver cancer	Liver cancer	Arm1 (*Lactobacillus* rhamnosus Probio-M9 + PD-1 Inhibitors) vs. Arm2 (placebo + PD-1 Inhibitors)	Phase 1	30 December 2023
NCT05094167	Recruiting	*Lactobacillus* Bifidobacterium V9 (Kex02) improving the efficacy of carilizumab combined With platinum in non-small cell lung cancer patients	Non-small Cell Lung Cancer	Arm1 (*Lactobacillus* Bifidobacterium V9 + Carilizumab Combined With Platinum) vs. Arm2 (placebo + Carilizumab Combined With Platinum)	Not Applicable	30 December 2023
NCT03686202	Recruiting	Feasibility study of microbial ecosystem therapeutics (MET-4) to evaluate effects of fecal microbiome in patients on immunOtherapy (MET4-IO)	All Solid Tumors	Arm1 (MET-4 strains + immune checkpoint inhibitors) vs. Arm2 (immune checkpoint inhibitors)	Early Phase 1	1 December 2023
NCT05220124	Recruiting	A study of probiotics administration in the immunotherapy of urothelial bladder carcinoma	Bladder Urothelial Carcinoma	Arm1 [Live Combined (Bifidobacterium, *Lactobacillus* and *Enterococcus* Capsules) + Immunotherapy] vs. Arm2 (Immunotherapy)	Phase 4	30 November 2024

## Discussion

With the increased use of ICIs in tumor therapy, tremendous effort has been made to uncover possible factors that affect their efficacy. An increasing amount of research indicates that the gut microbiome plays a critical role among these identified factors (Christofi et al., 2019). Whether probiotics can improve the response to ICIs in tumor patients is still being debated. For all we know, this is the first meta-analysis to investigate the relationship between probiotics and ICI efficacy in cancer patients. We synthesized all the available evidence and found a trend for probiotic use to prolong PFS and increase DCR, although it was of borderline statistical significance. We also found that probiotics were significantly positively correlated with OS and ORR in cancer patients administrated with ICIs. More importantly, our results also showed that NSCLC patients treated with ICIs in combination with probiotics would achieve significantly longer OS and PFS, as well as higher ORR and DCR.

The mechanisms by which probiotics promote the efficacy of ICIs have been thoroughly investigated in several animal studies. Back in 2015, Sivan et al. (2015) reported that *Bifidobacterium* can promote dendritic cell (DC) function and T cell-directed antitumor immunity, thereby enhancing the efficacy of ICIs in a tumor-bearing murine model. Zhuo and colleagues discovered that *Lactobacillus acidophilus* lysates boosted CTLA-4 antitumor efficacy in mouse models, which was linked to increased CD8^+^ T cells, increased effector memory T cells (CD44^+^ CD8^+^ CD62L^+^), decreased Treg (CD4^+^ CD25^+^ Foxp3^+^) and M2 macrophages (F4/80^+^ CD206^+^) in the tumor microenvironment (Zhuo et al., 2019). They also found that *Lactobacillus acidophilus* lysates had an immunomodulatory impact on inhibition of the M2 polarization and the IL-10 expressed levels of LPS-activated macrophages (Zhuo et al., 2019). The clinical practice has found that some cancer patients treated with ICIs have a history of antibiotic administration, and the meta-analysis found that the use of antibiotics may be related to worse outcomes in cancer patients treated with ICIs (Xu et al., 2020; Yang et al., 2020; Yu et al., 2021). Thus, the use of antibiotics may lead to a reduction in the abundance of the above-mentioned probiotics, which may reduce the efficacy of the ICIs.

Gao et al. (2021) found that *Lactobacillus rhamnosus* Probio-M9 administration can improve the effect of anti-PD-1 antitumor therapy by restoring the antibiotic-disrupted gut microbiome. *Lactobacillus rhamnosus* GG has also been found to enhance the anti-tumor activity of anti-PD-1 by increasing tumor-infiltrating DCs and T cells. Mechanistically, *Lactobacillus rhamnosus* GG alone or in combination with an anti-PD-1 antibody triggered type I interferon production in DCs, which enhanced the cross-initiation of anti-tumor CD8^+^ T cells (Si et al., 2022). Furthermore, due to the effect of *bifidobacterium* on regulatory CD4^+^ cells, whose metabolic and immunological suppressive functions are altered, this probiotic can rescue mice from an otherwise fatal inflammatory syndrome caused by anti–CTLA-4 (Wang et al., 2018; Sun et al., 2020). These theories support our conclusion. Probiotic supplementation is a potentially promising means of improving the efficacy of ICIs. While most drugs have side effects and are sometimes harmful to the patient, probiotics have few side effects (Forslund et al., 2015; Kim et al., 2017; Scharping et al., 2017; Wu et al., 2017; Cha et al., 2018). Thus, doctors can easily prescribe probiotics for their patients. The results of our study have important implications for clinicians involved in the treatment of NSCLC.

This article has some inherent restrictions, to be sure. To begin with, this study was essentially a meta-analysis that relied on previously published articles. We did not have sufficient data to perform subgroup analyses based on the type of probiotics, type of ICIs, duration of use, etc. Secondly, we were unable to investigate the correlation between probiotic use and ICI-induced adverse events, which should be highlighted further in future research. Therefore, future larger, multi-institutional studies with standardized prospective data collection are needed to further confirm our findings above.

## Conclusion

Current evidence reveals that probiotics can improve the efficacy of ICI treatment in patients with NSCLC.

## Data Availability

The original contributions presented in the study are included in the article/Supplementary Material, further inquiries can be directed to the corresponding authors.

## Supplementary material

Supplementary Figure S1 Sensitivity analysis of progression-free survival. CL, Confidence interval; Univariate analysis (Dizman et al., 2022); Multivariable analysis (Svaton et al., 2020; Tomita et al., 2020; Spencer et al., 2021; Takada et al., 2021).

Supplementary Figure S2 Sensitivity analysis of overall survival. CL, confidence interval; Univariate analysis (Dizman et al., 2022); Multivariable analysis (Svaton et al., 2020; Tomita et al., 2020; Takada et al., 2021).

Supplementary Figure S3 Sensitivity analysis of the objective response rate. CL, confidence interval; Univariate analysis (Tomita et al., 2020; Dizman et al., 2022); Multivariable analysis (Miura et al., 2021; Takada et al., 2021).

Supplementary Figure S4 Sensitivity analysis of the disease control rate. CL, Confidence interval; Univariate analysis (Tomita et al., 2020; Dizman et al., 2022); Multivariable analysis (Spencer et al., 2021; Takada et al., 2021).

Supplementary Table S1 Detailed search strategies.
