# Supplementary figures and images for: The correlation between probiotic use and outcomes of cancer patients treated with immune checkpoint inhibitors
Source: Front Pharmacol. 2022 Aug 30;13:937874. doi: 10.3389/fphar.2022.937874 (PMC9468893; doi:10.3389/fphar.2022.937874)

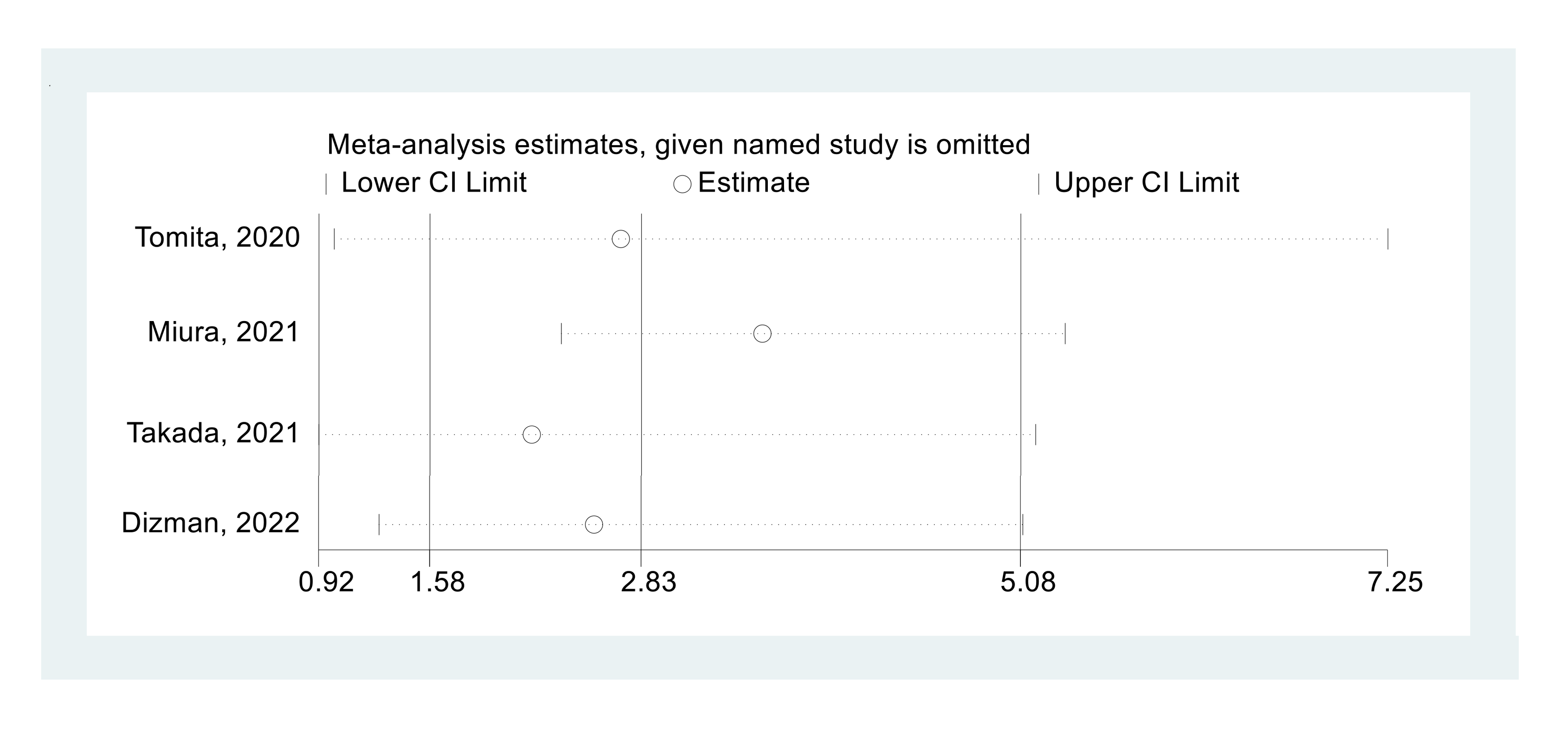

Supplement: Supplementary file 1 [file Image3.TIF]

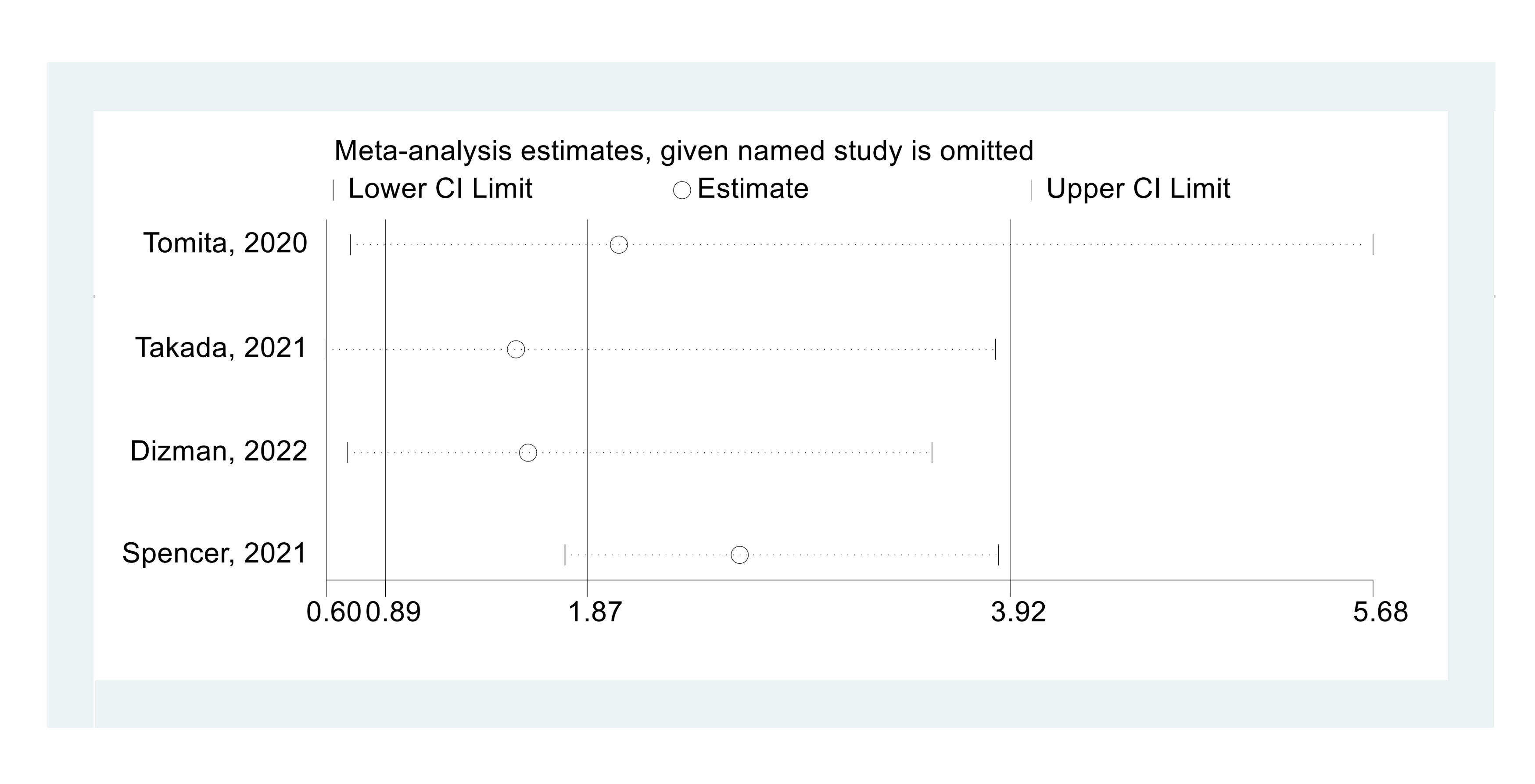

Supplement: Supplementary file 2 [file Image4.TIF]

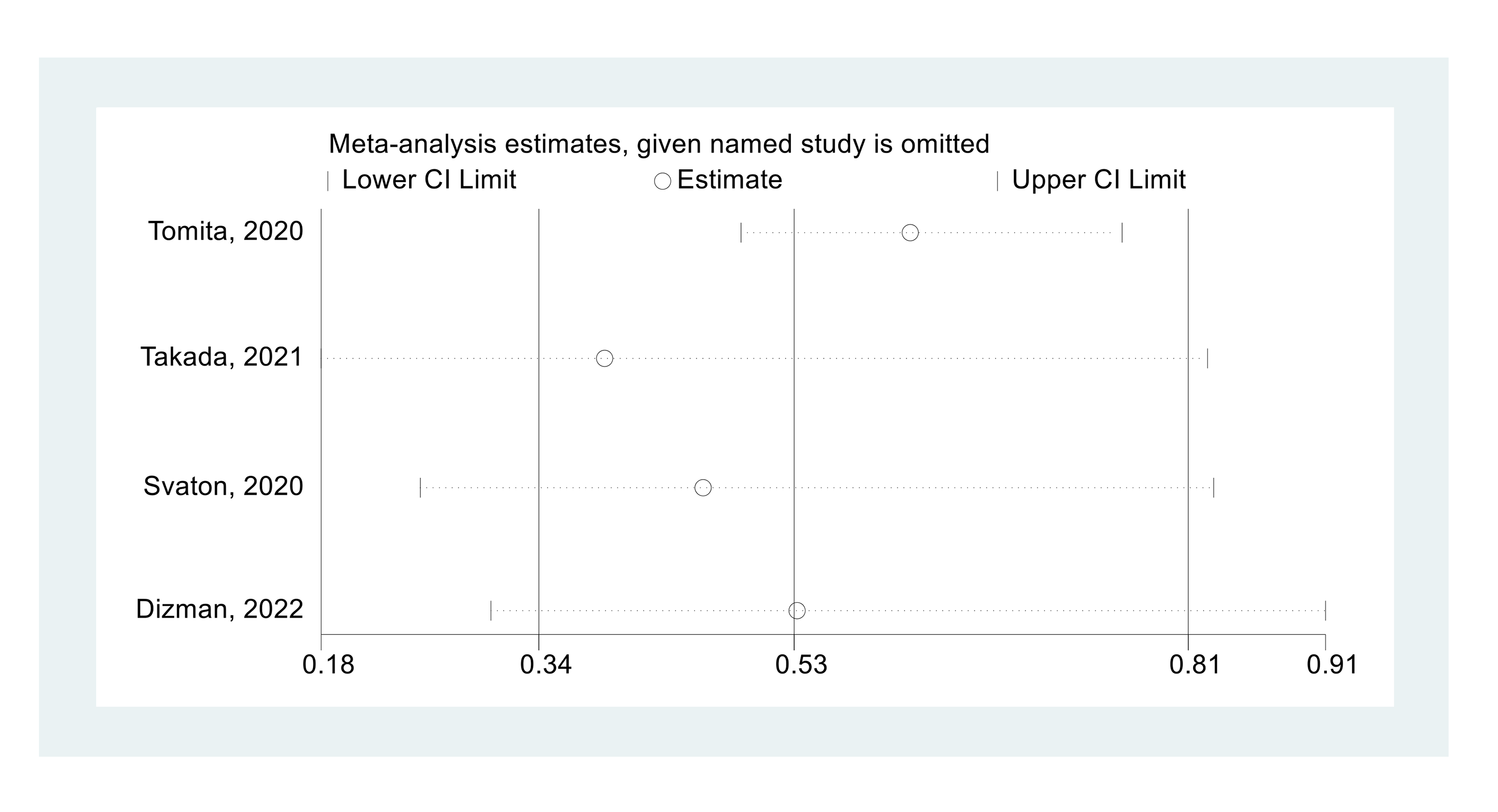

Supplement: Supplementary file 3 [file Image2.TIF]

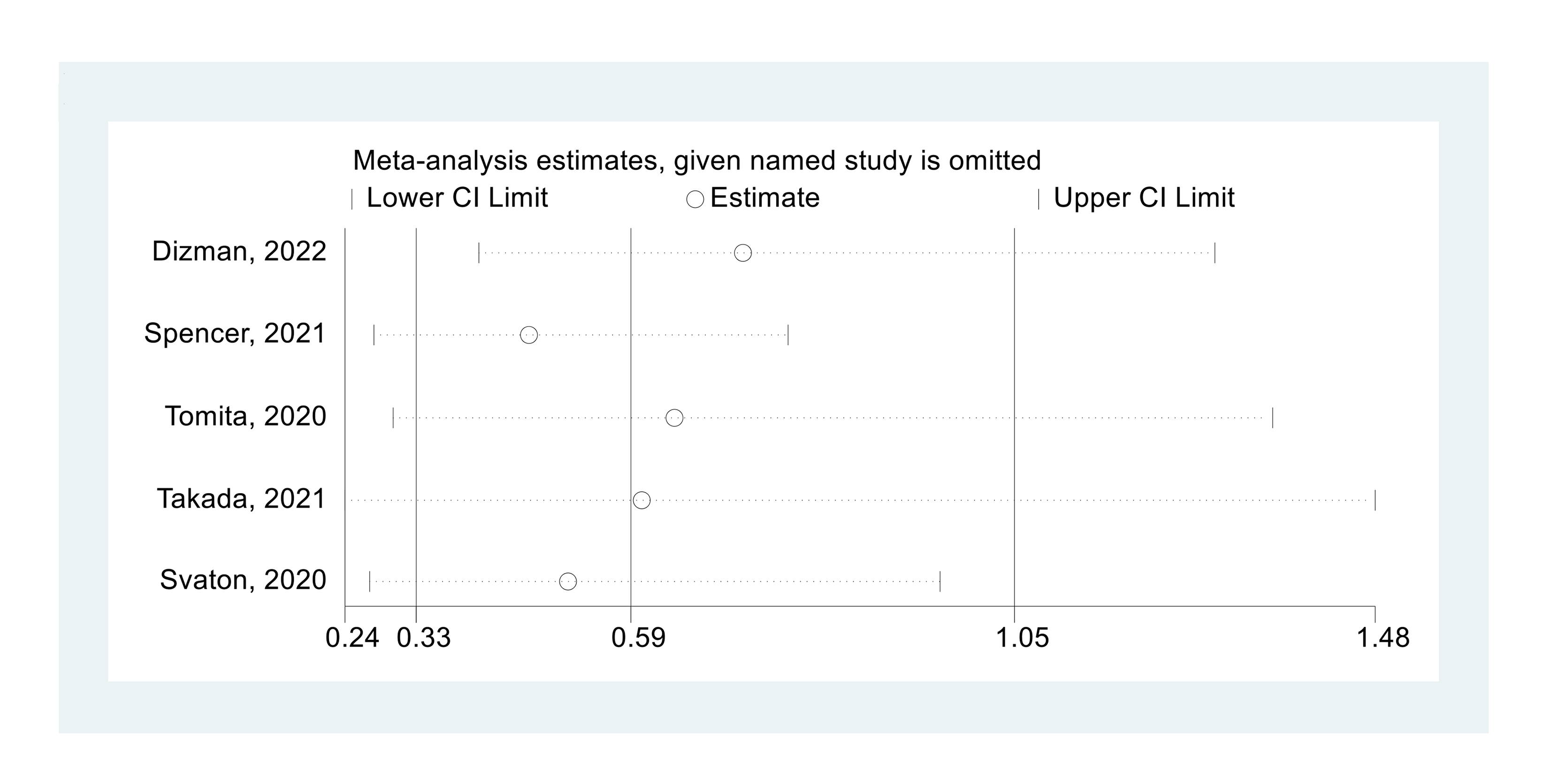

Supplement: Supplementary file 4 [file Image1.TIF]
