# Supplementary material for: The correlation between probiotic use and outcomes of cancer patients treated with immune checkpoint inhibitors
Source: Front Pharmacol. 2022 Aug 30;13:937874. doi: 10.3389/fphar.2022.937874 (PMC9468893; doi:10.3389/fphar.2022.937874)
Supplement: Supplementary file 5 [file DataSheet1.docx]

1. **Search strategies in Pubmed**

((((live bacterial supplementation) OR (Probiotics)) OR (Probiotic)) OR ("Probiotics"[Mesh])) AND (((((((((((((((((((((((((((((((((((((Immune Checkpoint Inhibitors) OR (Checkpoint Inhibitors, Immune)) OR (Immune Checkpoint Inhibitor)) OR (Checkpoint Inhibitor, Immune)) OR (Immune Checkpoint Blockers)) OR (Checkpoint Blockers, Immune)) OR (Immune Checkpoint Blockade)) OR (Checkpoint Blockade, Immune)) OR (Immune Checkpoint Inhibition)) OR (Checkpoint Inhibition, Immune)) OR (PD-L1 Inhibitors)) OR (PD L1 Inhibitors)) OR (PD-L1 Inhibitor)) OR (PD L1 Inhibitor)) OR (Programmed Death-Ligand 1 Inhibitors)) OR (Programmed Death Ligand 1 Inhibitors)) OR (PD-1-PD-L1 Blockade)) OR (Blockade, PD-1-PD-L1)) OR (PD 1 PD L1 Blockade)) OR (CTLA-4 Inhibitors)) OR (CTLA 4 Inhibitors)) OR (CTLA-4 Inhibitor)) OR (CTLA 4 Inhibitor)) OR (Cytotoxic T-Lymphocyte-Associated Protein 4 Inhibitors)) OR (Cytotoxic T Lymphocyte Associated Protein 4 Inhibitors[Title/Abstract])) OR (Cytotoxic T-Lymphocyte-Associated Protein 4 Inhibitor)) OR (Cytotoxic T Lymphocyte Associated Protein 4 Inhibitor)) OR (PD-1 Inhibitors)) OR (PD 1 Inhibitors)) OR (PD-1 Inhibitor)) OR (Inhibitor, PD-1)) OR (PD 1 Inhibitor)) OR (Programmed Cell Death Protein 1 Inhibitor)) OR (Programmed Cell Death Protein 1 Inhibitors)) OR (ICI)) OR (ICIs)) OR ("Immune Checkpoint Inhibitors"[Mesh]))

1. **Search strategies in Embase**

#1 'probiotic agent'/exp

#2 probiotic OR probiotics OR (live AND bacterial AND supplementation)

#3 #1 OR #2

#4 'immune checkpoint inhibitor'/exp

#5 checkpoint AND inhibitors, AND immune OR (immune AND checkpoint AND inhibitor) OR (checkpoint AND inhibitor, AND immune) OR (immune AND checkpoint AND blockers ) OR (checkpoint AND blockers, AND immune) OR (immune AND checkpoint AND blockade) OR (checkpoint AND blockade, AND immune) OR (immune AND checkpoint AND inhibition) OR (checkpoint AND inhibition, AND immune) OR ('pd l1' AND inhibitors) OR (pd AND l1 AND inhibitors) OR ('pd l1' AND inhibitor) OR (pd AND l1 AND inhibitor) OR (programmed AND 'death ligand' AND 1 AND inhibitors) OR (programmed AND death AND ligand AND 1 AND inhibitors) OR ('pd 1 pd l1' AND blockade) OR (blockade, AND 'pd 1 pd l1') OR (1 AND pd AND l1 AND blockade) OR ('ctla 4' AND inhibitors) OR (ctla AND 4 AND inhibitors) OR ('ctla 4' AND inhibitor) OR (ctla AND 4 AND inhibitor) OR (cytotoxic AND 't lymphocyte associated' AND protein AND 4 AND inhibitors) OR (cytotoxic AND t AND lymphocyte AND associated AND protein AND 4 AND inhibitors) OR (cytotoxic AND 't lymphocyte associated' AND protein AND 4 AND inhibitor) OR (cytotoxic AND t AND lymphocyte AND associated AND protein AND 4 AND inhibitor) OR ('pd 1' AND inhibitors) OR (pd AND 1 AND inhibitors) OR ('pd 1' AND inhibitor) OR (inhibitor, AND 'pd 1') OR (pd AND 1 AND inhibitor) OR (programmed AND cell AND death AND protein AND 1 AND inhibitor) OR (programmed AND cell AND death AND protein AND 1 AND inhibitors)

#6 #4 OR #5

#7 #3 AND #6

1. **Search strategies in Cochrane**

#1 MeSH descriptor: [Immune Checkpoint Inhibitors] explode all trees

#2 (Checkpoint Inhibitors, Immune) OR (Immune Checkpoint Inhibitor) OR (Immune Checkpoint Blockers) OR (Immune Checkpoint Blockade) OR (Immune Checkpoint Inhibition)

#3 (PD-L1 Inhibitors) OR (PD L1 Inhibitors) OR (PD-L1 Inhibitor) OR (PD L1 Inhibitor) OR (Programmed Death-Ligand 1 Inhibitors)

#4 (Programmed Death Ligand 1 Inhibitors) OR (CTLA-4 Inhibitors) OR (CTLA 4 Inhibitors)

#5 (CTLA-4 Inhibitor) OR (CTLA 4 Inhibitor) OR (Cytotoxic T-Lymphocyte-Associated Protein 4 Inhibitors) OR (Cytotoxic T Lymphocyte Associated Protein 4 Inhibitors) OR (Cytotoxic T-Lymphocyte-Associated Protein 4 Inhibitor)

#6 (Cytotoxic T Lymphocyte Associated Protein 4 Inhibitor) OR (PD-1 Inhibitor) OR (Inhibitor, PD-1) OR (PD 1 Inhibitor) OR (Programmed Cell Death Protein 1 Inhibitor)

#7 (PD-1 Inhibitors) OR (PD 1 Inhibitors) OR (Programmed Cell Death Protein 1 Inhibitors)

#8 #1 OR #2 OR #3 OR #4 OR #5 OR #6 OR #7

#9 MeSH descriptor: [Probiotics] explode all trees

#10 (Probiotics) OR (Probiotics) OR (Probiotic) OR (live bacterial supplementation)

#11 #9 OR #10

#12 #8 AND #11
